# Supplementary material for: The Impact of Oxford Nanopore Technologies Based Methodologies on the Genome Sequencing and Assembly of Romanian Strains of Drosophila suzukii
Source: Insects. 2024 Dec 24;16(1):2. doi: 10.3390/insects16010002 (PMC11766098; doi:10.3390/insects16010002)
Supplement: Supplementary file 1 [file insects-16-00002-s001.zip › Supplementary_Figures_v23dec2024.pdf]

Supplementary figures

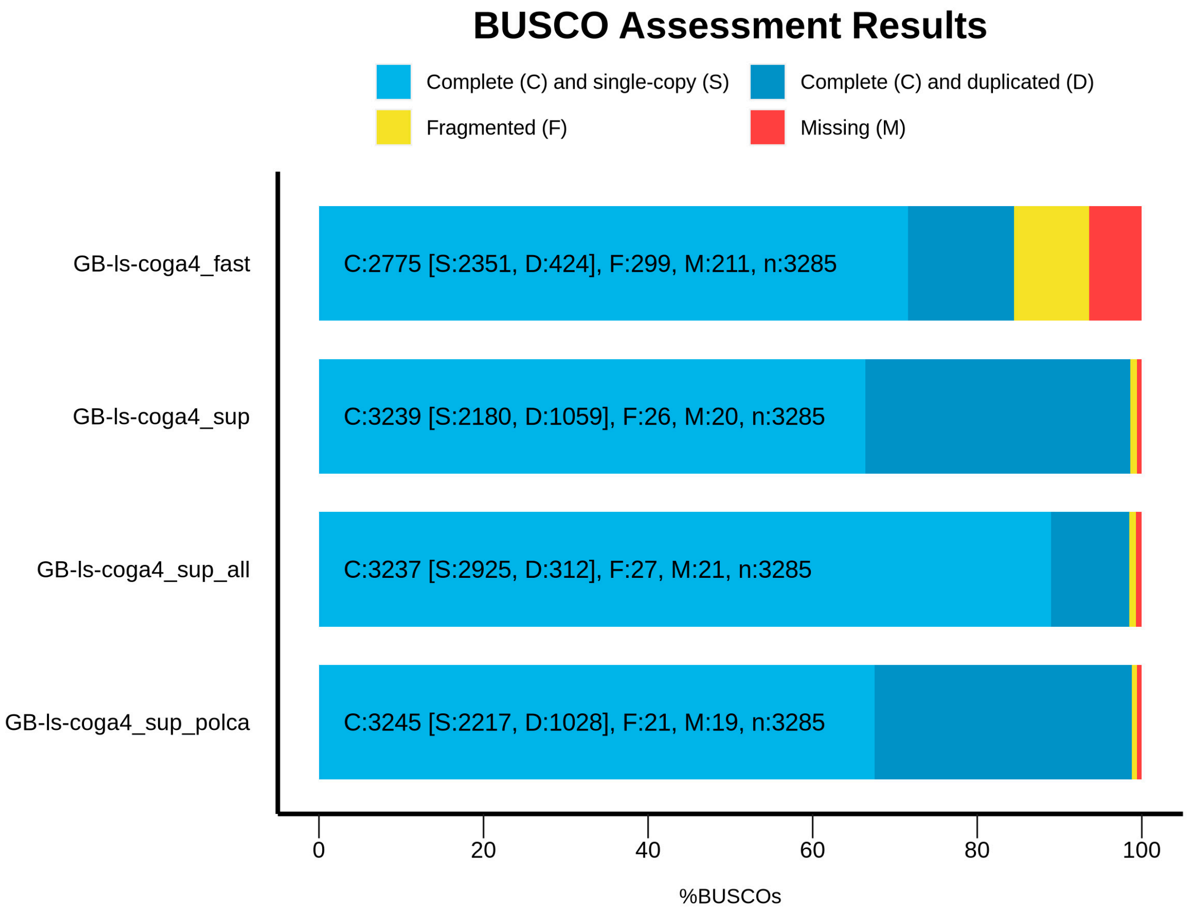

Supplementary Figure S1. The BUSCO results for the four tested assemblies. The assemblies designated as GB-ls-coga4_all and GB-ls-coga4_polca are referred to here as GB-ls-coga4_sup_all and GB-ls-coga4_sup_polca, respectively. It is apparent that GB-ls-coga4_all have better characteristics.

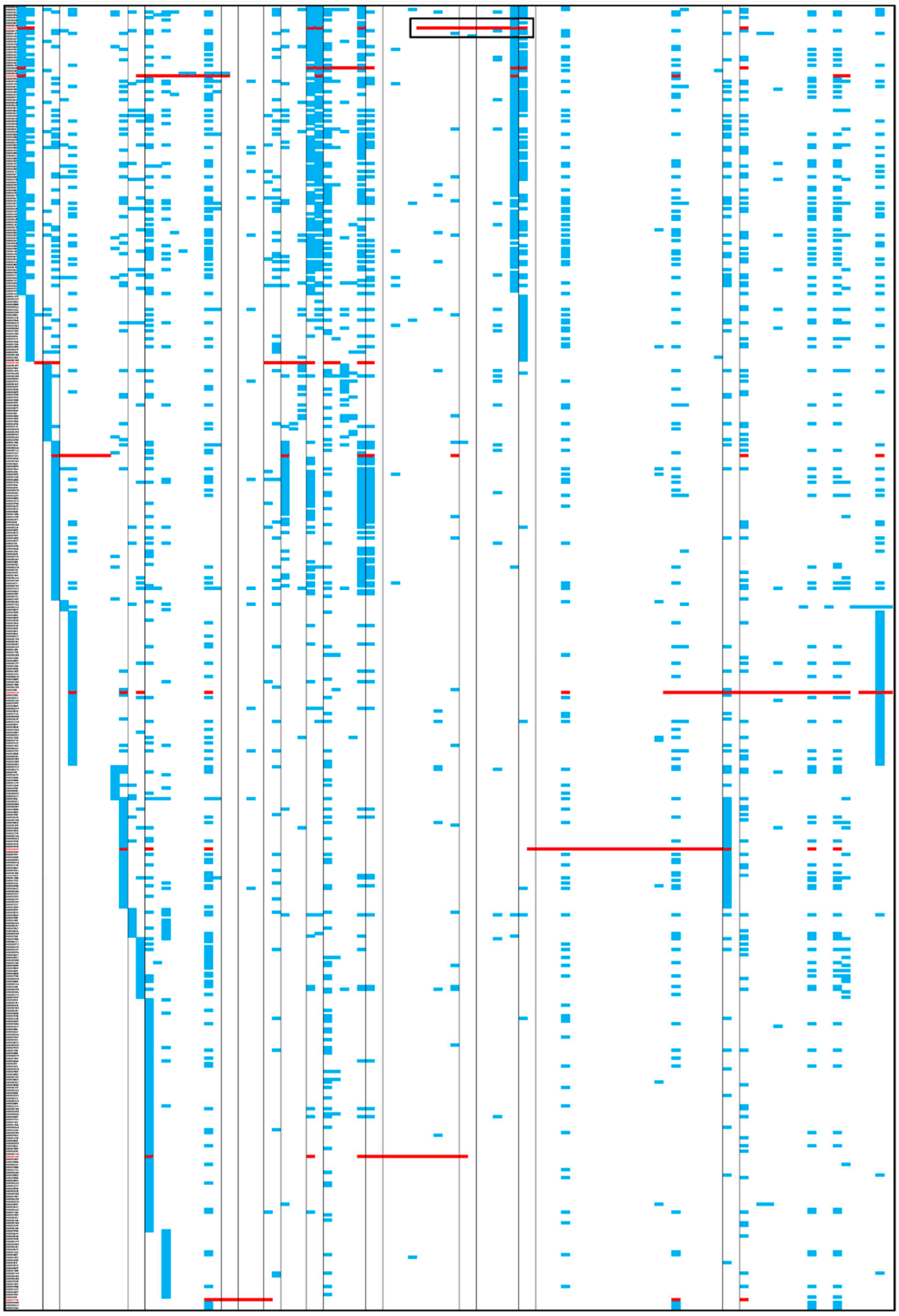

Supplementary Figure S2. The NW_023496809.1 scaffold from 2R has a highly repetitive nature, which is supported by the abundant blue hits generated when GB-ls-coga4_all is evaluated. Based on gene-queries, only five contigs containing overlapping alignments can be found. When ranked-queries strategy is used a more complex picture is apparent, as presented in the figure. Nine contigs can assure the whole scaffold linear coverage, including the tig00001376 contig (engulfed in the black rectangle) that is the only one that can be identified with the gene-queries strategy. The grey vertical lines are inserted in order to ease the identification of neighboring or overlapped consecutive contigs. Figure edits were created with BioRender.com.
