# Supplementary material for: The Impact of Oxford Nanopore Technologies Based Methodologies on the Genome Sequencing and Assembly of Romanian Strains of Drosophila suzukii
Source: Insects. 2024 Dec 24;16(1):2. doi: 10.3390/insects16010002 (PMC11766098; doi:10.3390/insects16010002)
Supplement: Supplementary file 1 [file insects-16-00002-s001.zip › Supplementary_Tables_v23dec2024.pdf]

Supplementary tables

Supplementary Table S1. Various sequencing parameters characterizing the sequencing outputs obtained for GB-ls-coga4 and ICDPP-ams-1 lines. Abbreviations for reads and bases refer to their corresponding number (k = kilo, 10^3, M = Mega, 10^6, and G = giga, 10^9). "Number of bases" and "N50 value" columns refer to nucleotide bases (b), and file sizes are shown in gigabytes (GB).

D. suzukii line	Flow cell	Input DNA concentration (fmoles)	Reads count	Number of Bases	N50 value	File sizes
GB-ls-coga4	FLO-MIN114	16.18	4.6 M	16.59 Gb	8.9 kb	198.7 GB
	FLO-FLG114	7.7	116.59 k	328.25 Mb	9.7 kb	3.8 GB
		7.7	76.72 k	242.47 Mb	10.09 kb	2.8 GB
		19.41	224.54 k	347.58 Mb	3.67 kb	4.2 GB
		20	34.94 k	87.12 Mb	7.3 kb	1.0 GB
		20	155.39 k	268.84 Mb	4.04 kb	3.1 GB
ICDPP-ams-1	FLO-MIN114	27.5	4.84 M	13.23 Gb	5.51 kb	155.5 GB
	FLO-FLG114	18	150.52 k	389.11 Mb	7 kb	4.5 GB
		18	143.84 k	361.56 Mb	6.9 kb	4.3 GB
		10.785	188.54 k	557.65 Mb	9.12 kb	6.12 GB
		10.785	166.87 k	517.41 Mb	9.81 kb	5.65 GB
		14.29	29.68 k	95.4 Mb	9.83 kb	1.04 GB

Supplementary Table S2. Results of one-sample t-tests performed on sequencing data for both GB4-ls-coga4 and ICDPP-ams-1 lines. Statistical testing was performed between data corresponding to five FLO-FLG114 and the single value corresponding to the FLO-MIN114. The CI abbreviation refers to the confidence intervals.

D. suzukii line	Statistical parameter	Input DNA concentration (fmoles)	Log_2 reads count	Log_2 number of Bases	N50 value	File sizes
GB4-ls-coga4	t value	-0.411	-11.914	-16.99	-1.431	-353.583
	p value	0.702	2.843×10^{-4}	7.037×10^{-5}	0.226	3.838×10^{-10}
	degrees of freedom	4	4	4	4	4
	sample mean	14.962	16.635	27.779	6960	2.98
	reference mean	16.18	22.133	33.949	8900	198.7
	lower 95% CI	6.725	15.353	26.771	3195.351	1.443
	upper 95% CI	23.199	17.916	28.788	10724.649	4.517
ICDPP-ams-1	t value	-8.137	-10.93	-11.527	4.589	-170.088
	p value	1.241×10^{-3}	3.979×10^{-4}	3.234×10^{-4}	1.012×10^{-2}	7.167×10^{-9}
	degrees of freedom	4	4	4	4	4
	sample mean	14.372	16.813	28.295	8532	4.322
	reference mean	27.5	22.207	33.623	5510	155.5
	lower 95% CI	9.892	15.443	27.011	6703.517	1.854
	upper 95% CI	18.852	18.183	29.57823	10360.482	6.789

Supplementary Table S3. Reads quality details for GB4-ls-coga4 and ICDPP-ams-1 *D. suzukii* lines and each sequencing experiment. Data is based on NanoPlot reads quality evaluation. Dorado basecalling models are noted fast for dna_r10.4.1_e8.2_400bps_fast@v4.3.0 and sup for dna_r10.4.1_e8.2_400bps_sup@v4.3.0. The Mb abbreviation refers to Mega (10^6) nucleotide bases. Q value represents the quality Phred score.

D. suzukii line	Flow cell	Dorado basecalling model	Mean read length	Median read length	Mean read quality (Q value)	Median read quality (Q value)	Percent of reads with Q > 7	Percent of reads with Q > 10	Percent of reads with Q > 15 (number of reads)
GB-ls-coga4	FLO-MIN114	fast	3736.3	1476	8.3	9.9	86.3%	47.6%	0% (510)
		sup	3751.8	1469	12.8	18.0	92.9%	87.8%	71.9%
	FLO-FLG114	fast	3243.7	968	8.6	9.5	94.8%	29.6%	0% (7)
		sup	3319.6	984	14.6	16.8	99.0%	96.6%	72.2%
		fast	3625.1	1166	8.3	9.5	90.7%	32.2%	0% (4)
		sup	3720.0	1185	13.0	16.3	96.1%	91.4%	62.9%
		fast	1656.9	652	7.9	9.2	85.9%	27.2%	0% (19)
		sup	1700.5	669	12.4	16.0	94.2%	88.4%	59.8%
		fast	2759.9	983	8.1	9.6	86.6%	37.8%	0% (2)
		sup	2829.8	1000	12.0	16.0	92.5%	87.2%	59.6%
		fast	1923.9	780	9.2	10.2	95.7%	57.3%	0% (61)
		sup	1956.4	789	15.2	18.0	98.5%	96.4%	81.7%
ICDPP-ams-1	FLO-MIN114	fast	2733.6	1292	8.3	10.3	83.6%	55.7%	0.1% (5640)
		sup	2756.1	1298	11.7	17.1	90.3%	84.1%	65.3%
	FLO-FLG114	fast	2783.7	1020	8.6	9.7	91.6%	40%	0% (46)
		sup	2816.3	1032	13.5	16.4	97.7%	92.6%	64.3%
		fast	2517.7	917	7.8	9.3	85.4%	29.1%	0% (51)
		sup	2546.7	925	11.7	15.5	92.7%	87.1%	56.5%
		fast	3155.5	1071	8.4	9.6	90.7%	38.3%	0% (45)
		sup	3197.1	1079	13.3	16.3	97.0%	92.5%	63.1%
		fast	3306.6	1110	8.0	9.2	86.1%	27.3%	0% (13)
		sup	3360.2	1122	12.1	15.1	95.4%	87.7%	50.8%
		fast	3323.8	1105	8.2	9.4	91.1%	28.8%	0.1% (18)
		sup	3370.7	1116	12.6	15.8	96.3%	93.0%	60.1%

Supplementary Table S4. Results of one-sample t-tests performed on sequencing data for the GB4-ls-coga4 line and basecalled with the Dorado dna_r10.4.1_e8.2_400bps_fast@v4.3.0. Statistical testing was performed between data corresponding to five FLO-FLG114 and the single value corresponding to the FLO-MIN114. The CI abbreviation refers to the confidence intervals. Q value represents the quality Phred score.

D. suzukii line	Statistical parameter	Mean read length	Median read length	Mean read quality (Q value)	Median read quality (Q value)	log_2 reads with Q > 7	log_2 reads with Q > 10	log_2 reads with Q > 15
GB4-ls-coga4	t value	-2.91	-6.376	0.529	-1.826	-11.57	-12.258	-6.696
	p value	4.367×10^{-2}	3.103×10^{-3}	0.625	0.142	3.19×10^{-4}	2.543×10^{-4}	2.587×10^{-3}
	degrees of freedom	4	4	4	4	4	4	4
	sample mean	2641.9	909.8	8.42	9.6	16.497	15.144	3.197
	reference mean	3736.3	1476	8.3	9.9	21.921	21.061	8.994
	lower 95%CI	1597.772	663.264	7.79	9.144	15.196	13.804	0.794
	upper 95%CI	3686.028	1156.336	9.049	10.056	17.799	16.485	5.601

Supplementary Table S5. Results of one-sample t-tests performed on sequencing data for the GB4-ls-coga4 line and basecalled with the Dorado dna_r10.4.1_e8.2_400bps_sup@v4.3.0. Statistical testing was performed between data corresponding to five FLO-FLG114 and the single value corresponding to the FLO-MIN114. The CI abbreviation refers to the confidence intervals. Q value represents the quality Phred score.

D. suzukii line	Statistical parameter	Mean read length	Median read length	Mean read quality (Q value)	Median read quality (Q value)	log_2 reads with Q > 7	log_2 reads with Q > 10	log_2 reads with Q > 15
GB4-ls-coga4	t value	-2.705	-6.064	1.025	-3.683	-11.66	-11.522	-11.254
	p value	5.379×10^{-2}	3.735×10^{-3}	0.363	2.115×10^{-2}	3.09×10^{-4}	3.239×10^{-4}	3.552×10^{-4}
	degrees of freedom	4	4	4	4	4	4	4
	sample mean	2705.26	925.4	13.44	16.62	16.588	16.524	16.062
	reference mean	3751.8	1469	12.8	18	22.052	21.969	21.682
	lower 95%CI	1631.211	676.496	11.707	15.579	15.287	15.212	14.675
	upper 95%CI	3779.309	1174.304	15.173	17.66	17.889	17.836	17.449

Supplementary Table S6. Results of paired two-sample t-test achieved on sequencing data for the GB4-ls-coga4 line. Statistical testing was performed between data corresponding to the Dorado dna_r10.4.1_e8.2_400bps_fast@v4.3.0 and dna_r10.4.1_e8.2_400bps_sup@v4.3.0 basecalling models. Logarithmic transformations were performed for variables that contained multiple degrees of magnitude between measurements. The CI abbreviation refers to the confidence intervals. Q value represents the quality Phred score.

Data	Parameter	t value	p value	Lower 95% CI	Upper 95% CI
Untransformed	Mean read length	-4.549	6.117×10^{-3}	-86.678	-24.088
	Median read length	-2.943	0.032	-22.167	-1.499
	Mean read quality (Q value)	-13.91	3.45×10^{-5}	-5.845	-4.022
	Median read quality (Q value)	-26.958	1.314×10^{-6}	-7.886	-6.513
	Percent of reads with Q > 7	-7.105	8.557×10^{-4}	-7.535	-3.531
	Percent of reads with Q > 10	-11.139	1.017×10^{-4}	-64.841	-40.525
	Number of reads with Q > 7	-1.119	0.314	-227053.1	89306.059
	Number of reads with Q > 10	-1.22	0.277	-1170122	416879.725
	Number of reads with Q > 15	-1.152	0.301	-2037210	776423.989
$\log_2$ transformed	$\log_2$ mean read length	-5.761	2.213×10^{-3}	-0.042	-0.016
	$\log_2$ median read length	-3.303	0.021	-0.035	-0.004
	$\log_2$ number of reads with Q > 7	-6.764	1.073×10^{-3}	-0.134	-0.06
	$\log_2$ number of reads with Q > 10	-7.769	5.653×10^{-4}	-1.731	-0.871
	$\log_2$ number of reads with Q > 15	-32.718	5.012×10^{-7}	-13.844	-11.827

Supplementary Table S7. Results of one-sample t-tests performed on sequencing data for the ICDPP-ams-1 line and basecalled with the Dorado dna_r10.4.1_e8.2_400bps_fast@v4.3.0. Statistical testing was performed between data corresponding to five FLO-FLG114 and the single value corresponding to the FLO-MIN114. The CI abbreviation refers to the confidence intervals. Q value represents the quality Phred score.

D. suzukii line	Statistical parameter	Mean read length	Median read length	Mean read quality (Q value)	Median read quality (Q value)	log_2 reads with Q > 7	log_2 reads with Q > 10	log_2 reads with Q > 15
ICDPP-ams-1	t value	1.794	-6.927	-0.707	-9.274	-10.889	-11.259	-18.566
	p value	0.147	2.279×10^{-3}	0.518	7.52×10^{-4}	4.04×10^{-4}	3.545×10^{-4}	4.953×10^{-5}
	degrees of freedom	4	4	4	4	4	4	4
	sample mean	3017.46	1044.6	8.2	9.44	16.654	15.192	4.912
	reference mean	2733.6	1292	8.3	10.3	21.965	21.379	12.461
	lower 95%CI	2578.068	945.443	7.807	9.182	15.299	13.666	3.783
	upper 95%CI	3456.852	1143.757	8.593	9.697	18.008	16.717	6.041

Supplementary Table S8. Results of one-sample t-tests performed on sequencing data for the ICDPP-ams-1 line and basecalled with the Dorado dna_r10.4.1_e8.2_400bps_sup@v4.3.0. Statistical testing was performed between data corresponding to five FLO-FLG114 and the single value corresponding to the FLO-MIN114. The CI abbreviation refers to the confidence intervals. Q value represents the quality Phred score.

D. suzukii line	Statistical parameter	Mean read length	Median read length	Mean read quality (Q value)	Median read quality (Q value)	log_2 reads with Q > 7	log_2 reads with Q > 10	log_2 reads with Q > 15
ICDPP-ams-1	t value	1.859	-6.717	2.741	-5.252	-10.751	-10.868	-11.292
	p value	0.136	2.557×10^{-3}	5.185×10^{-2}	6.289×10^{-3}	4.24×10^{-4}	4.068×10^{-4}	3.505×10^{-4}
	degrees of freedom	4	4	4	4	4	4	4
	sample mean	3058.2	1054.8	12.64	15.82	16.765	16.683	16.059
	reference mean	2756.1	1298	11.7	17.1	22.084	21.982	21.617
	lower 95%CI	2606.952	954.281	11.688	15.143	15.391	15.329	14.692
	upper 95%CI	3509.448	1155.319	13.592	16.497	18.138	18.037	17.426

Supplementary Table S9. Results of paired two-sample Student's t-test performed on sequencing data for the ICDPP-ams-1 line. Statistical testing was performed between data corresponding to the Dorado dna_r10.4.1_e8.2_400bps_fast@v4.3.0 and dna_r10.4.1_e8.2_400bps_sup@v4.3.0 basecalling models. Logarithmic transformations were performed for variables that contained multiple degrees of magnitude between measurements. The CI abbreviation refers to the confidence intervals. Q value represents the quality Phred score.

Data	Parameter	t value	p value	Lower 95% CI	Upper 95% CI
Untransformed	Mean read length	-7.887	5.268×10^{-4}	-49.987	-25.413
	Median read length	-9.271	2.455×10^{-4}	-12.134	-6.866
	Mean read quality (Q value)	-17.75	1.041×10^{-5}	-4.884	-3.649
	Median read quality (Q value)	-45.049	1.018×10^{-7}	-6.818	-6.082
	Percent of reads with Q > 7	-11.922	7.319×10^{-5}	-8.286	-5.347
	Percent of reads with Q > 10	-10.181	1.568×10^{-4}	-66.339	-39.593
	Number of reads with Q > 7	-1.179	0.291	-21272.65	78957.784
	Number of reads with Q > 10	-1.347	0.236	-874068.3	272824.609
	Number of reads with Q > 15	-1.154	0.3	-1943878	739044.437
log_2 transformed	log_2 mean read length	-11.37	9.207×10^{-5}	-0.022	-0.014
	log_2 median read length	-8.489	3.728×10^{-4}	-0.017	-0.009
	log_2 number of reads with Q > 7	-11.308	9.453×10^{-3}	-0.138	-0.087
	log_2 number of reads with Q > 10	-7.899	5.23×10^{-4}	-1.781	-0.906
	log_2 number of reads with Q > 15	-21.79	3.778×10^{-6}	-12.091	-9.539
