# Supplementary figures and images for: The Impact of Oxford Nanopore Technologies Based Methodologies on the Genome Sequencing and Assembly of Romanian Strains of Drosophila suzukii
Source: Insects. 2024 Dec 24;16(1):2. doi: 10.3390/insects16010002 (PMC11766098; doi:10.3390/insects16010002)

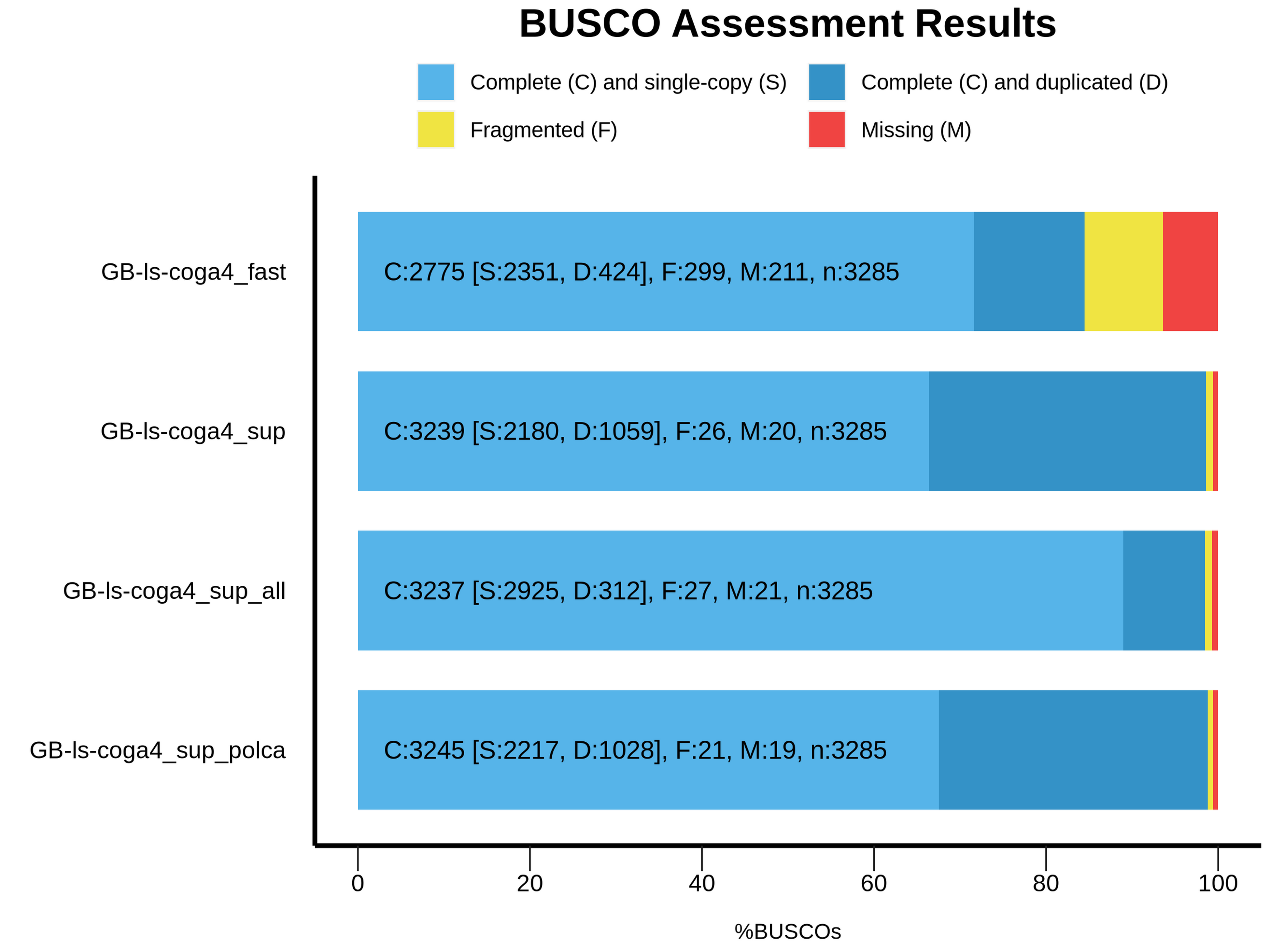

Supplement: Supplementary file 1 [file insects-16-00002-s001.zip › Figure S1_busco_analysis.png]

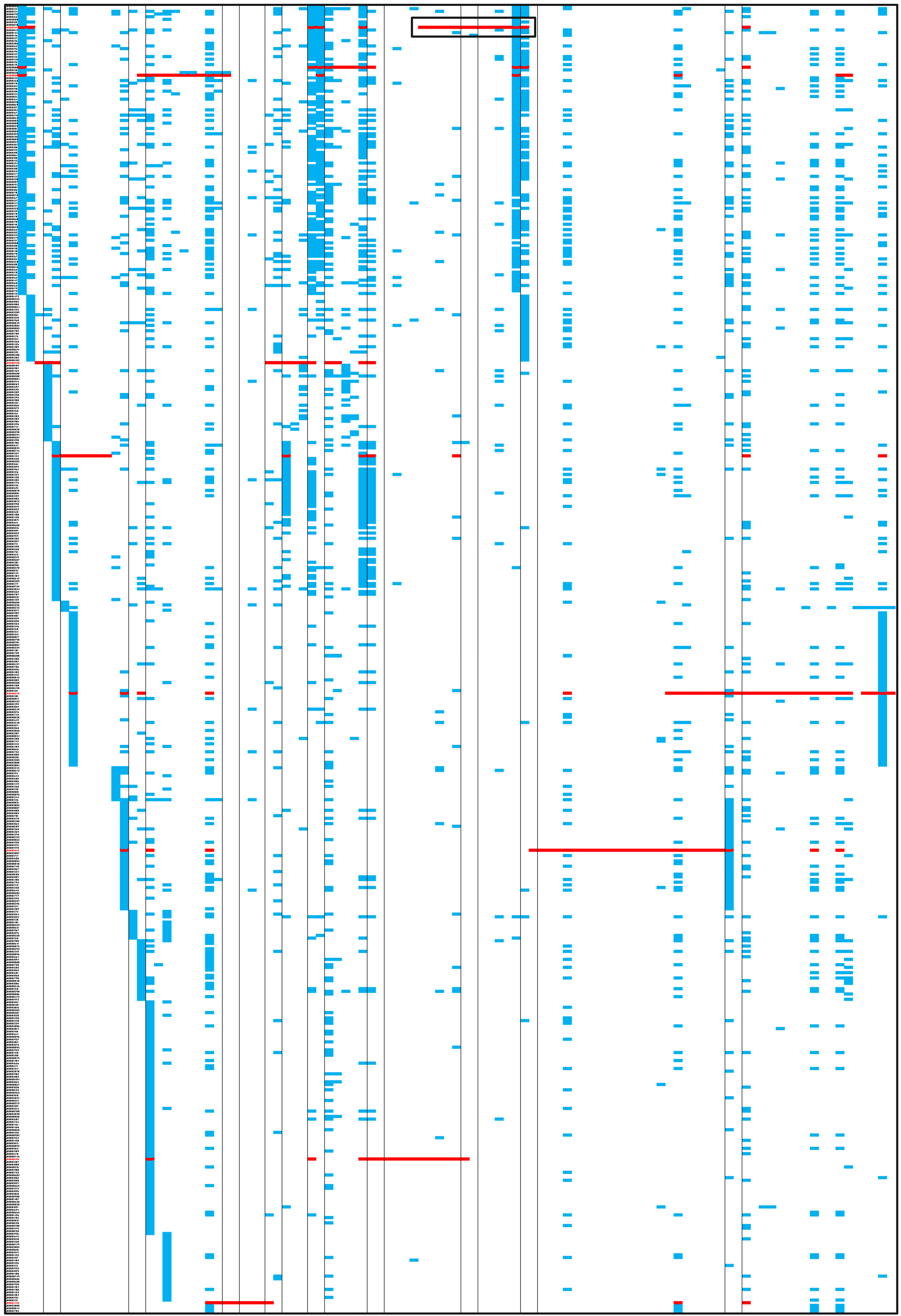

Supplement: Supplementary file 1 [file insects-16-00002-s001.zip › Figure S2_ranked_queries_strategy.png]
